# An embedded intracranial seizure monitor for objective outcome measurements and rhythm identification

**DOI:** 10.1109/EMBC40787.2023.10340850

**Published:** 2023-07

**Authors:** John E. Fleming, Moaad Benjaber, Robert Toth, Mayela Zamora, Kei Landin, Ali Kavoosi, Jonathan Ottoway, Tom Gillbe, Rory J. Piper, Tara Noone, Hannah Campbell, Ivor Gillbe, Marios Kaliakatsos, Martin Tisdall, Antonio Valentín, Timothy Denison

**Affiliations:** 1 MRC Brain Network Dynamics Unit, Nuffield Department of Clinical Neurosciences, University of Oxford, Oxford OX1 3TH, UK; 2 Institute of Biomedical Engineering, Department of Engineering Sciences, University of Oxford, Oxford OX3 7DQ, UK; 3 Bioinduction Ltd., Bristol, UK; 4 Great Ormond Street Institute of Child Health, University College London, London WC1N 1EH, UK; 5 Department of Neurosurgery, Great Ormond Street Hospital, London WC1N 3JH, UK; 6 Department of Basic and Clinical Neuroscience, King’s College London, London SE5 9RT, UK

## Abstract

Providing clinicians with objective outcomes of neuromodulation therapy is a key unmet need, especially in emerging areas such as epilepsy and mood disorders. These diseases have episodic behavior and circadian/multidien rhythm characteristics that are difficult to capture in short clinical follow-ups. This work presents preliminary validation evidence for an implantable neuromodulation system with integrated physiological event monitoring, with an initial focus on seizure tracking for epilepsy. The system was developed to address currently unmet requirements for patients undergoing neuromodulation therapy for neurological disorders, specifically the ability to sense physiological data during stimulation and track events with seconds-level granularity. The system incorporates an interactive software tool to enable optimal configuration of the signal processing chain on an embedded implantable device (the Picostim–DyNeuMo Mk-2) including data ingestion from the device loop recorder, event labeling, generation of filter and classification parameters, as well as summary statistics. When the monitor parameters are optimized, the user can wirelessly update the system for chronic event tracking. The simulated performance of the device was assessed using an *in silico* model with human data to predict the real-time device performance at tracking recorded seizure activity. The *in silico* performance was then compared against its performance in an *in vitro* model to capture the full environmental constraints such as sensing during stimulation at the tissue electrode interface. *In vitro* modeling demonstrated comparable results to the *in silico* model, providing verification of the software tool and model. This study provides validation evidence of the suitability of the proposed system for tracking longitudinal seizure activity. Given its flexibility, the event monitor can be adapted to track other disorders with episodic and rhythmic symptoms represented by bioelectrical behavior.

## I Introduction

Epilepsy is a neurological disorder characterized by a predisposition to have epileptic seizures – the symptoms of abnormal excessive or synchronous neuronal activity in the brain [1]. Epilepsy affects more than 65 million people worldwide, and has been estimated to cost the United States economy alone 12.5 billion dollars annually [2]. Antiseizure medications are the first-line treatment option for epilepsy; however, approximately one third of patients do not respond to these medications and may benefit from alternative therapies [3].

Deep brain stimulation (DBS) is an intracranial neuromodulation therapy that is emerging as an alternative option for the treatment of patients with drug-resistant epilepsy [4]. DBS delivers direct electrical stimulation to key intracranial targets with the aim of preventing the generation and propagation of seizure activity in the brain. Market-available DBS device capabilities are currently confined to using either ‘open-loop’ stimulation (the Medtronic Percept™), where the device provides continuous or duty (on-off) cycles of stimulation at fixed parameters, or responsive stimulation (the Neuropace RNS™), where stimulation is triggered in response to measured epileptiform activity. These devices currently do not have the capabilities to detect and record seizure activity in real time. At present, longitudinal sensing on the Medtronic Percept™ is limited to a data logger that tracks the 10 minute average power within a specified frequency band at 10 minute intervals. In contrast, sensing capabilities on the Neuropace RNS™ are turned off when stimulation is being delivered by the device.

The clinical benefits of simultaneous sensing and stimulation are twofold. Firstly, real-time seizure-onset detection allows for adaptive stimulation to be delivered to abort breakthrough seizure activity. Secondly, the ability to longitudinally detect and record a patient’s seizure activity offers clinicians objective data with regard to seizure frequency, seizure rhythms, and, critically, the impact of interventions such as stimulation regime alterations.

Current clinical trials evaluating the efficacy of DBS therapies for epilepsy are dependent on patient/carer manual diaries, which are notoriously inaccurate due to noncompliance and unawareness of seizure activity [5]. There is a clear need to embed seizure detection capabilities into next-generation DBS devices for the treatment of patients with epilepsy. Recorded signal features such as line-length or spectral power can be utilized to train DBS device-embedded classifiers for the online detection of seizure events as a surrogate to patient-reported seizure diaries [6], [7], [8]. At present, however, there are several technical issues that may hamper the implementation of these devices as reliable seizure monitors. These issues include 1) resolving microvolt level signals during stimulation therapy [9], [10], 2) incorporation of diurnal and other rhythmic variations in epileptic brain patterns [11] and 3) accommodating the heterogeneity of epileptic patient recorded brain activity.

**Fig. 1:**
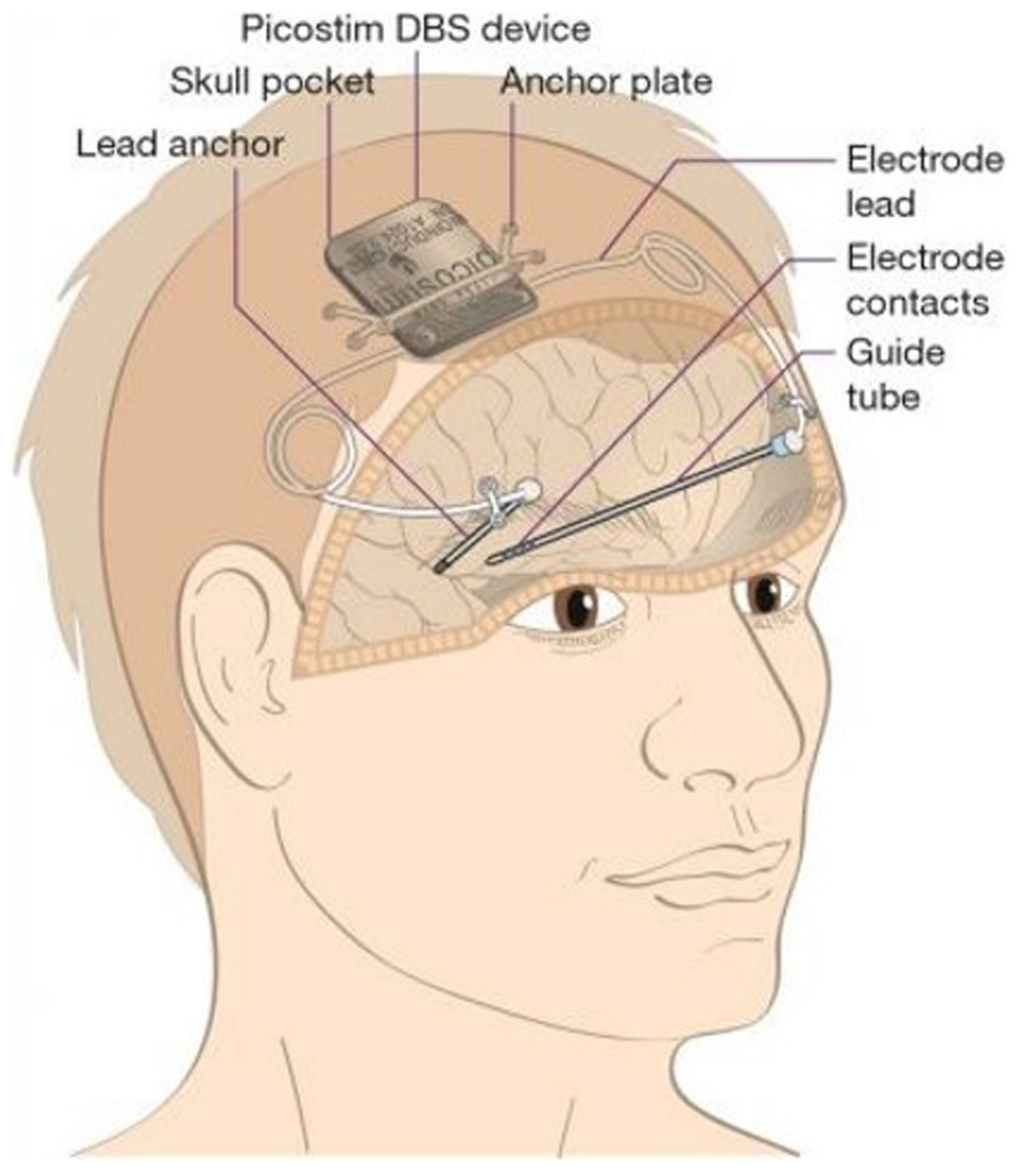
Illustration of the cranially-mounted Picostim–DyNeuMo neuromodulation research system.

In this study we build on the work of the Picostim–DyNeuMo research system, presented in [9], [12], to enable seizure detection and recording embedded on the neuromodulation device, Fig. 1. We developed an algorithm workflow to facilitate device configuration for patient-specific seizure activity monitoring. The workflow enabled preliminary *in silico* prediction of the seizure monitor performance prior to real-time investigation of the detector embedded on the neuromodulation device in an *in vitro* test-bench.

## II Materials and Methods

### A Next-Generation Neuromodulation Device Requirements

Next-generation neuromodulation devices must enable the objective assessment of neuromodulation therapies. To this end, capabilities required include 1) maintenance of physiological data recording capabilities during stimulation, 2) recording of longitudinal data with sufficient granularity to observe typical events (e.g. seconds for thalamic seizures) and 3) enable flexible algorithm design for long-term deployment in a patient-specific or disease-specific manner.

### B Picostim-DyNeuMo Overview

The Picostim–DyNeuMo research system is a cranially-mounted, rechargeable neuromodulation device that enables dynamic variation of delivered stimulation programs in response to movement (Mk-1) [12], time (Mk-1&2), and sensed biopotential signals (Mk-2) [9]. These embedded device capabilities are enabled via an onboard device accelerometer, clock and filters that facilitate configurable algorithm deployment for a variety of neurological indications.

### C OxCAT Overview

**Fig. 2:**
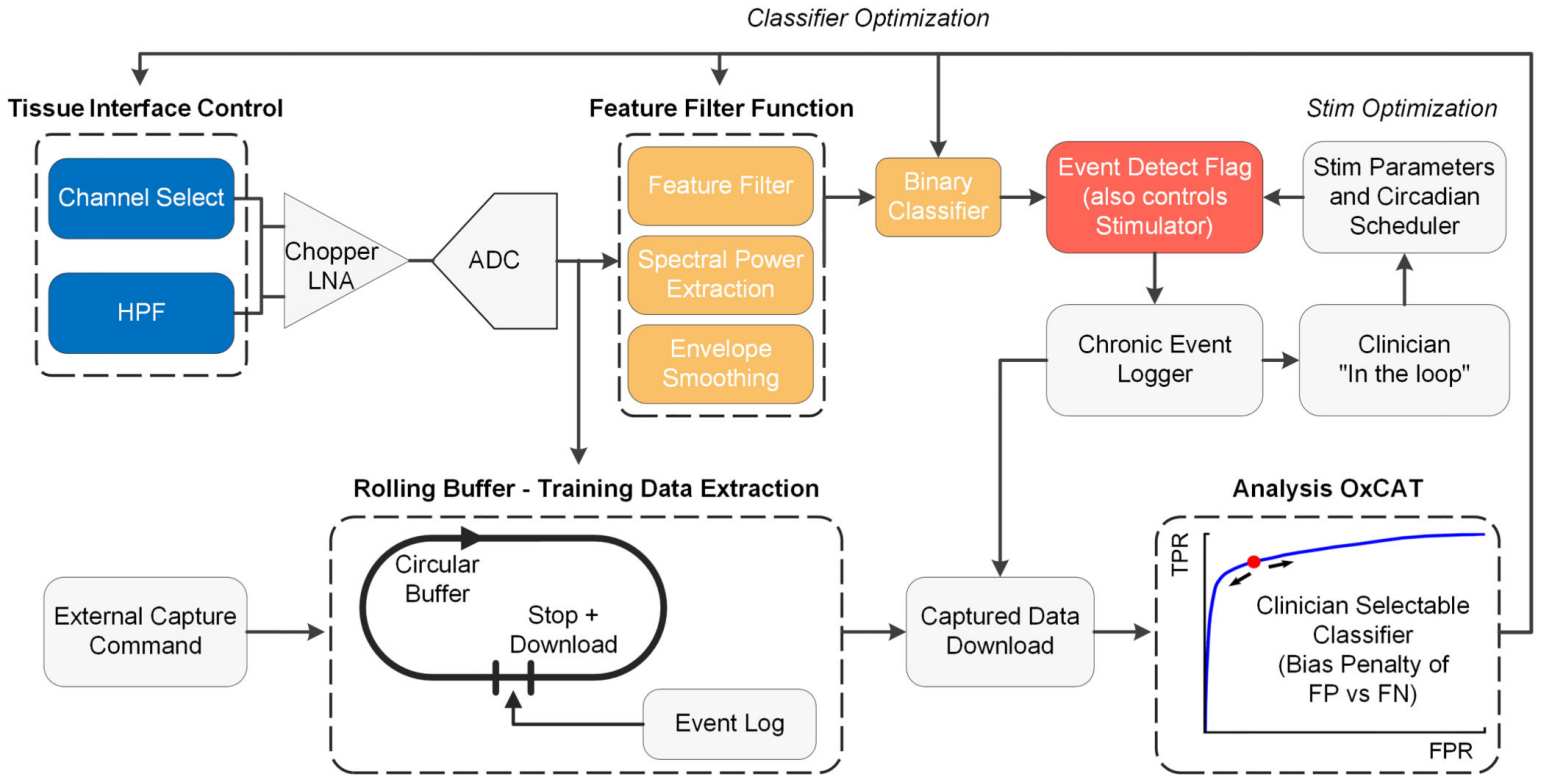
Overview of the Picostim-DyNeuMo Mk-2 signal chain for data logging, classifier training and chronic event tracking. Patient-specific device configuration was enabled by the OxCAT workflow.

The Oxford Configurable Algorithm Tool (OxCAT) is a software workflow that was developed to facilitate flexible configuration of the embedded signal processing chain and classifier on the DyNeuMo Mk-2 device. The OxCAT workflow was developed as a Jupyter Notebook using Python 3.8. The workflow enables 1) ingestion of pre-recorded data as an input to the workflow, 2) annotation of the input data, 3) a power spectral density viewer for annotated data segments, 4) a filter design tool for dynamic configuration of the device signal processing chain and 5) an interactive receiver-operating characteristic (ROC) curve viewer for dynamic selection of the embedded classifier threshold. In addition, the OxCAT simulates operation of the device signal processing chain and classifier to provide a preliminary prediction of the system performance with the specified configuration. If suitable performance is predicted, the workflow provides the ability to export the specified device configuration as a YAML file for upload onto the DyNeuMo Mk-2 device for real-time operation. An overview of the DyNeuMo Mk-2 device signal chain and OxCAT workflow for device configuration are visualized in Fig. 2.

### D Data Overview

In this study, seizure data from two patients with genetic generalized epilepsy previously recorded by King’s College Hospital was utilized. Both patients were implanted with externalized DBS electrodes at the centromedian thalamus (CMT). The dataset for patient 1 was recorded in 2011, while patient 2’s dataset was recorded in 2014. The original datasets included both synchronized scalp electroencephalogram (EEG) and CMT LFP recordings; however, only the recorded EEG was utilized by a trained clinician to annotate the data for periods of electrographic seizure activity. Electrographic seizures were identified as epileptiform discharges which lasted greater than three seconds in duration. Discharge or spike activity less than three seconds was considered to be interictal epileptiform activity and was subsequently not included as annotated seizure events. Eight hours of data was analyzed from patient 1 and 69 seizure events were identified. For patient 2, 11 seizure events were identified from analyzing five hours and twelve minutes from their dataset.

Only the synchronized LFP recordings were utilized as input to the OxCAT workflow and saline tank test-bench. The LFP recordings were preprocessed by resampling the recorded signals to a 625 Hz sampling frequency. The data was then segmented into sections to extract each annotated seizure event, including the 30 seconds before and after. Finally, the segmented seizure data was concatenated together to create the final dataset, which was used as an input to the OxCAT workflow and saline tank test-bench.

During annotated seizure periods, patient 1 displayed a broadband increase in power between 0 – 22 Hz, where a ‘bump’ between 9 – 14 Hz was also observed. For this reason the bandpass filter in the DyNeuMo Mk-2 signal processing chain was centered on 11.5 Hz with low and high cutoff frequencies of 9 Hz and 14 Hz, respectively. Similarly, patient 2 displayed a broadband power increase in frequencies between 0 – 24 Hz, in addition to a ‘bump’ between 9 – 23 Hz. The bandpass filter for patient 2 was subsequently centered on 16.5 Hz with 14 Hz and 19 Hz bandpass cutoff frequencies. For both patient datasets, the highpass and lowpass (smoothing) filters in the signal processing chain were set to 6.8 Hz and 1 Hz, respectively.

### E OxCAT In Silico Test-Bench Overview

The performance of the classifier was simulated by filtering the input signal with the specified signal processing chain configured in the OxCAT workflow. To generate the classifier ROC curve, the true and false positive rates for the classifier were calculated by comparing the clinician-annotated data labels with the classifier-predicted labels on a sample-by-sample basis. When determining the overall classifier performance for detecting annotated seizure events, the true positives were defined as positive classifications that occurred within 3 seconds following a clinician-annotated label. False positives were similarly defined as positive classifications that were not associated with a clinician-annotated label in the 3 seconds prior to classification. The metrics calculated for determining overall classifier performance were precision, recall and F_1_ score.

For each dataset the classifier threshold for the device was selected from examination of the corresponding ROC curve in the OxCAT workflow. This was done in a patient-specific manner due to signal levels between patients varying as a result of interpatient variability. To meet the need for accurate seizure counting when implementing the system as a seizure burden monitor, we biased selection of an appropriate threshold to maximize classifier true positives, while minimizing classifier false positives. To this end, patient 1 was selected to have a threshold of 26.7 μV, while patient 2 was selected to have a threshold of 4.2 μV. For both classifier implementations the debounce period for stimulation state transitions on the device was fixed at 2 seconds, i.e. the minimum duration between successive classifications.

### F Adaptive DBS Test-Bench Overview

**Fig. 3:**
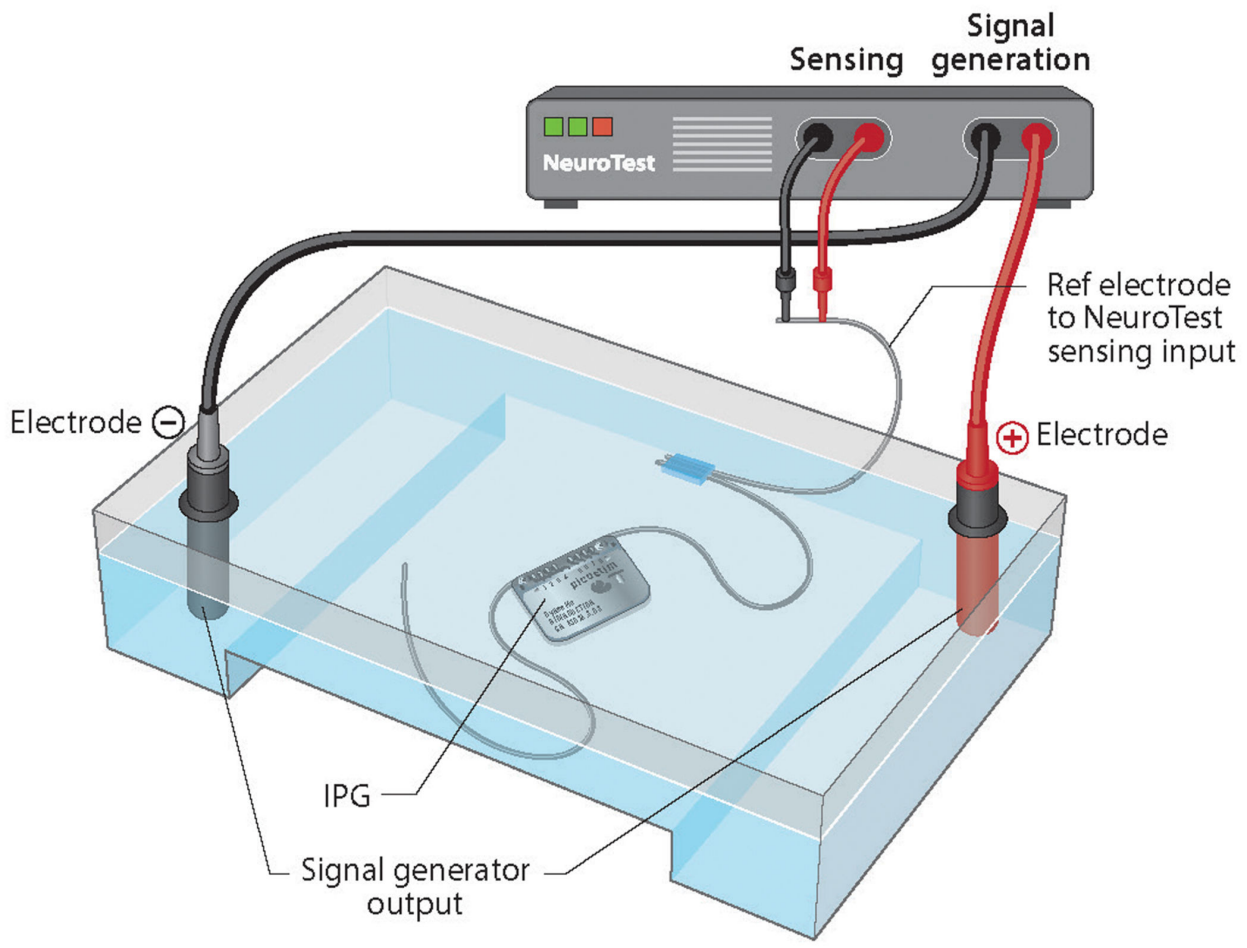
The *In vitro* test bench is comprised of the following 1) an electrophoresis tank filled with 0.9% saline, 2) the NeuroTest board, which is used to: 2.1) inject patient data, 2.2) calibrate signal levels, and 2.3) record the DyNeuMo Mk-2 system performance *in Vitro* using the reference electrode.

The adaptive DBS (aDBS) test-bench is a configurable *in vitro* test setup, designed to evaluate the performance of an implantable aDBS device in an environment that is representative of *in vivo* use cases. The test-bench is made from two main elements: 1) The saline tank: which is made using an electrophoresis system filled with a 0.9% saline solution that the investigated device was submerged in during testing. Using the saline solution allowed the test-bench to emulate the charge distribution of stimulation artifacts. Furthermore, this setup allows testing of the saline-electrode interface, which is similar to the tissue-electrode interface *in vivo*, and its effects on the sensing and stimulation performance of aDBS devices. 2) The NeuroTest board: a custom made hardware designed to test the performance of aDBS devices. The NeuroTest board has two main functions. a) NeuroTest-DAC: a two channel arbitrary voltage signal generator that can generate signals with a large dynamic range between 1 μV–1V (RMS). This allows the NeuroTest-DAC to replay prerecorded biopotential signals with amplitudes representative of real signals recorded *in vivo*. b) NeuroTest-DAQ: a two channel data acquisition system with a 24-bit resolution, with a noise floor of 1 μV RMS at 1 kHz bandwidth, and a configurable sampling frequency from 1 kHz to 16 kHz. During testing, an aDBS–configured device can be placed in the center of the saline tank with the DBS electrodes placed on either side of the device case. Reference DBS electrodes connected to the NeuroTest-DAQ are placed in parallel and in close proximity with the aDBS device electrodes under test. These reference electrodes are used to record the activity in the saline tank to evaluate the performance of the aDBS device. Finally, the NeuroTest-DAC is used to inject prerecorded biopotential signals across the saline tank for *in vitro* device testing. The NeuroTest has the ability to run a test and record the performance over 24 hours. This allows researchers and engineers to test their aDBS systems and algorithms for a duration that mimics real world use-cases. Fig 3 illustrates the *in vitro* test bench setup.

### G Picostim-DyNeuMo Mk-2 Seizure Monitor Setup

The DyNeuMo Mk-2 was configured to prototype the embedded seizure burden monitor proof-of-concept *in vitro*. During the *in vitro* test only the patient 1 dataset was selected as it contained the highest number of seizure events. After selecting the required classifier settings for patient 1 using the OxCAT workflow, the DyNeuMo Mk-2 was setup to act in seizure monitoring mode. To enable the seizure monitoring mode the DyNeuMo Mk-2 adaptive DBS mode was required. This was set as follows: 1) by uploading the classifier configuration YAML file to the DyNeuMo Mk-2 and 2) setting the stimulation programs; one for event and a second for non-events. For seizure diary mode both event and non-event stimulation programs should be set to the same settings. The DyNeuMo Mk-2 then logs each time there is a transition between event and non-event stimulation programs within a few seconds resolution. This ensures that the device is not changing the therapy for patients at this stage and only acting as an embedded seizure burden monitor. However, to assess the performance of the device *in vitro*, the DyNeuMo Mk-2 was set to perform in full adaptive mode, by setting two different stimulation programs with different current amplitudes. This makes testing the system performance easier by detecting the different stimulation levels in the test-bench, using the reference electrodes connected to NeuroTest board. The stimulation programs used during the test were 1) non-event program (P0): 1mA, 125 Hz, 90 μs, active recharge; 2) event program (P1): 3mA, 125 Hz, 90 μs active recharge.

## III Results

### A OxCAT Classifier Predicted Performance

**Fig. 4:**
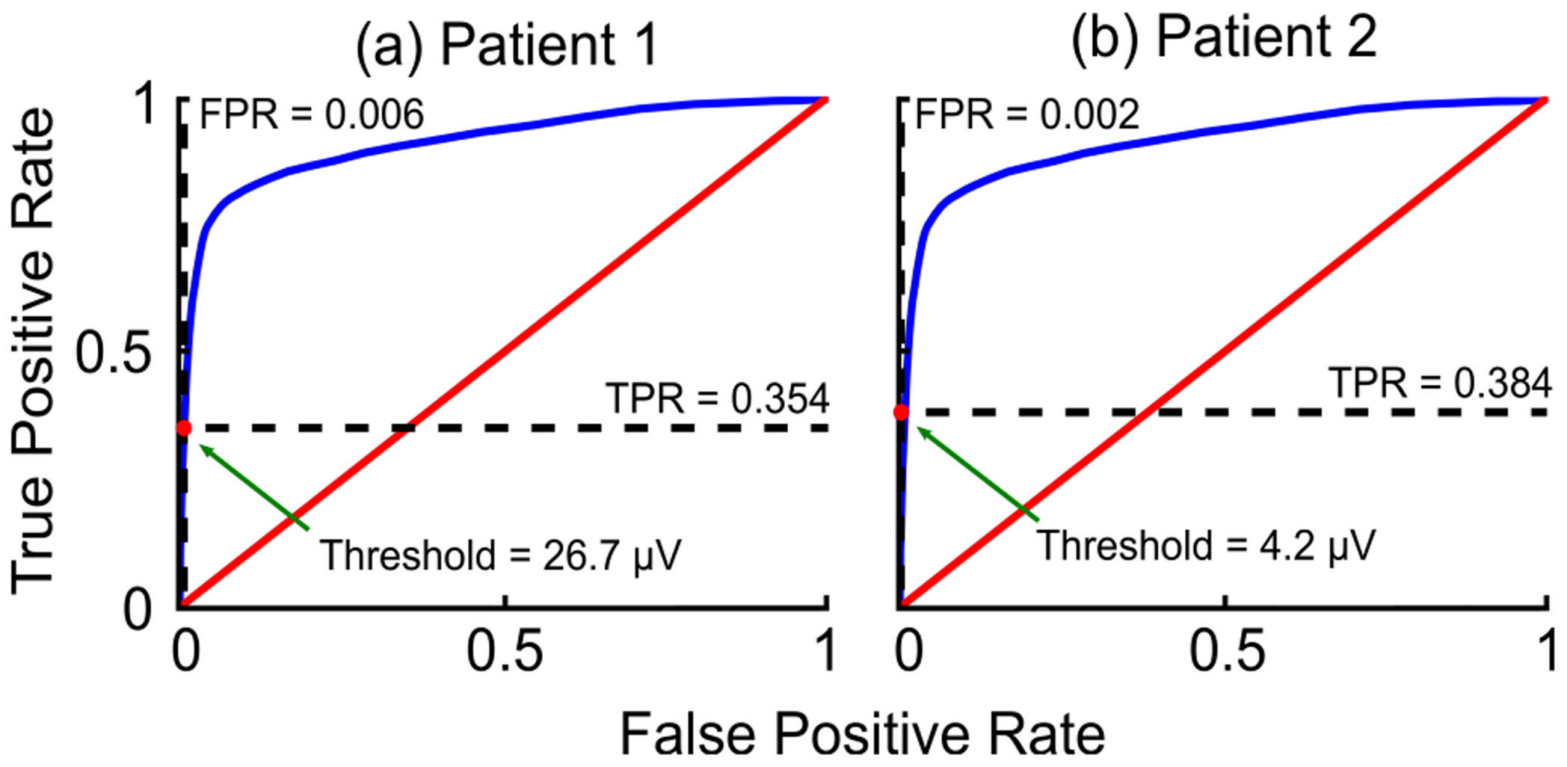
ROC curves generated from the OxCAT workflow for the DyNeuMo Mk-2 classifier. The red dot marks the selected operating point, with dashed lines indicates the corresponding TPR and FPR. Note: TPR and FPR were calculated on a sample-by-sample basis, while true / false positives and false negatives reported in the classifier summary statistics were calculated on an event-by-event basis (see Section II, D).

Classifiers configured by the OxCAT workflow demonstrated good predicted performance at tracking annotated seizure events. For each patient the classifier threshold was conservatively selected from the respective ROC curve to minimize the false positive rate, Fig. 4. The classifier performance was predicted to result in an F_1_ score of 86.1% and 88.0% for patients 1 and 2, respectively. Additional classifier performance summary statistics are included in Table I.

### B In Vitro Test Results

#### 1) Adaptive DBS Test-Bench Signal Levels

The NeuroTest board and *in vitro* test-bench setup was calibrated to replay the patient 1 data with signal levels within ±5% of original data signal levels when sensed by the DyNeuMo Mk-2 in saline. This was a crucial part of the test as a higher error in the replayed patient signal levels compared to the original dataset will result in a different amount of detected seizure events. The results of the achieved signal levels sensed by the DyNeuMo Mk-2 in the *in vitro* test-bench setup were comparable to the original signal levels recorded in the band of interest (7 – 15 Hz) for patient 1. These results are summarized in Table II.

#### 2) Picostim-DyNeuMo Mk-2 Performance

**Fig. 5:**
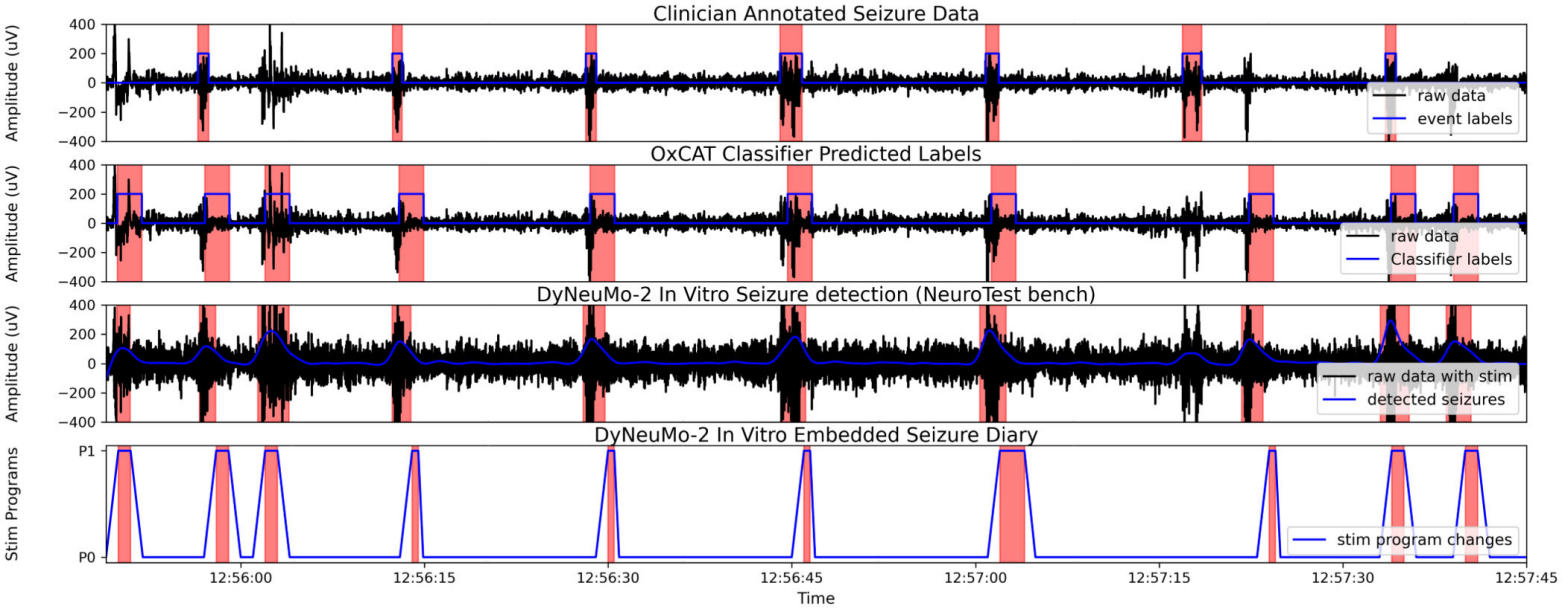
Performance of the DyNeuMo Mk-2 seizure detection during stimulation *in vitro*, compared to the estimated classifier output from OxCAT workflow, and the clinician annotated seizure data. Detected seizure events for the different tests are shown in red.

For patient 1 the *in vitro* test of the DyNeuMo Mk-2 achieved a 7.5% higher trigger rate than that estimated by the OxCAT workflow. Also, when compared to the clinician annotated events (as illustrated in Table III) the device trigger rate was higher by 4.3%. This was mainly due to the slightly larger signal level sensed by the DyNeuMo Mk-2 in saline compared to the original raw patient 1 dataset that was used to configure the classifier. This issue could have been resolved by increasing the classifier threshold to match the slight increase in the signal level. However no effect in the classifier’s performance to detect seizures was observed due to this when the DyNeuMo Mk-2 was running in full adaptive mode, switching between different stimulation current amplitudes (P0: 1mA and P1: 3mA). This was due to the synchronized sense and stimulation technique used in the DyNeuMo Mk-2 to mitigate stimulation artifact [13]. This technique enables DyNeuMo Mk-2 to detect 1 μV RMS signals in the presence of stimulation. Results of the first 120 seconds of seizure detection *in vitro* are illustrated in Fig. 5.

## IV Discussion

In this paper, we present the DyNeuMo Mk-2 and OxCAT workflow as a configurable system to enable patient-specific seizure detection. The OxCAT algorithm workflow enables flexible configuration of the DyNeuMo device for tracking power-in-band variations in recorded LFP data associated with seizures. The predicted behavior of the device classifier by the OxCAT workflow well-matched that of its *in vitro* implementation when the device was tested in a saline tank test-bench. This work provides preliminary evidence that the system can act as a surrogate to patient/carer-reported seizure diaries to facilitate the measurement of objective data for epilepsy. In addition, the configurability of the system is relevant for other disease states beyond epilepsy where power-in-band variations are identified as markers of disease symptoms. There has been much interest for instance in Parkinson’s disease where variations in beta-band (13 – 30 Hz) power recorded from the subthalamic nucleus has been identified as a biomarker for motor impairment symptoms of the disease [14]. The relatively small signal power in Parkinson’s field potentials makes the ability to sense microvolt-level signals in the presence of variable stimulation a key requirement to maintain visibility to physiological biomarkers.

Although this work provides preliminary evidence that the DyNeuMo device may function well for seizure detection, there are technical limitations of this work that should be highlighted. In relation to the device hardware, the classifier on the device is limited to a two second debounce period. This is to ensure that the device guarantees that patients will receive this minimum duration of clinical stimulation when stimulation on the device is varying. Second, although the DyNeuMo Mk-2 device enables deterministic, time-based feed-forward adjustments to the stimulation and classifier the data presented herein focuses primarily on the implementation of the system for daytime seizure monitoring. Additional data recorded over the entire 24-hour day-night cycle is required to enable classifier implementation for true diurnal configurations of the DyNeuMo Mk-2 device as a seizure monitor, where variations in frequency bands associated with sleep may lead to variation in classifier performance at night-time [15].

In addition to the technical limitations highlighted above there are several outstanding research questions that can affect implementation of the proposed seizure burden monitor. Firstly, the correlation between device-recorded electrographic seizure activity and the occurrence of clinical (patient-experienced) seizures needs to be further investigated. There are a variety of different seizure types and further investigation of how these seizure types relate to the data recorded from deep brain targets should be further assessed to ensure accurate tracking of specified seizure types when the monitor is deployed longitudinally. Furthermore, additional considerations should be taken when the proposed seizure monitor is deployed as part of stimulation therapy. For the presented datasets selection of the classifier threshold was biased to prioritize maximizing true positives for accurate seizure monitoring. In practice, the therapeutic trade-off between true and false positives should be carefully considered when implemented for stimulation therapy. For epilepsy, failure to deliver stimulation during seizure events may lead to worse therapeutic outcomes than falsely providing stimulation during non-seizure periods. In fact, there is increasing evidence that the therapeutic benefits of responsive neurostimulation are provided when stimulation is applied during non-seizure states, rather than seizure states [16]. Similar considerations should be taken when considering other neurological disorders where the therapeutic trade-off may differ in a disease-specific manner, potentially leading to worsened outcomes for patients’ daily activities. Moreover, care might be required to provide the appropriate phasing of adaptive stimulation in response to detected events.

The DyNeuMo Mk-2 has a rolling memory to store up to 1200 events, which is sufficient to record seizures over at least 24 hours for most patients. The DyNeuMo Mk-2 prioritizes critical operations, such as delivering stimulation first, and running the real-time classifier second. Therefore, the system only has a resolution of 1 – 2 second while recording event changes in the user logs (as illustrated in the bottom panel of Fig. 5).

Finally, the data presented in this first proof of concept is limited. Datasets used for classifier training were historically recorded using externalized DBS leads. For each patient, recordings were limited solely to the hospital period between implantation of the DBS leads and pulse generator. Longitudinal data collection from these patients for further analysis and device testing was thus not possible. The aim of this paper is to demonstrate that the DyNeuMo Mk-2 system is able to detect seizures in the presence of stimulation accurately, and as predicted by the OxCAT workflow, so that it can be deployed as a seizure burden monitor. In the future, the performance of the DyNeuMo Mk-2 classifier must be tested with additional data captured by the device embedded loop recorder.

## Figures and Tables

**Table I:** OxCAT Classifier Predicted Performance

Classifier Metrics	Patient 1	Patient 2
Clinician Annotated Events	69	11
Classifier Threshold (μV)	26.7	4.2
Classifier Triggers (Stim Triggers)	134	42
Precision (%)	86.8	78.6
Recall (%)	85.5	100.0
F_1_ Score (%)	86.1	88.0

Note: OxCAT workflow classifier triggers are equivalent to stimulation program transitions in the DyNeuMo device.

**Table II:** Test-bench calibration

RMS value in first 4 events (BW 7 – 15 Hz)	Original dataset (μV RMS)	DyNeuMo Mk-2 sensed data (μV RMS)	Error %
Event 1	31.07	29.65	-4.5
Event 2	45.25	46.22	2.1
Event 3	36.46	36.71	0.7
Event 4	32.21	32.41	0.6

**Table III:** Validation of Algorithm Triggering

Annotation	OxCAT Estimate	Recorded
138	134	144

## Data Availability

The authors will consider requests to access the data in a trusted research environment.

## Clinical relevance

An implantable neuromodulation system is presented that enables chronic tracking of physiological events in disease. This physiological monitor provides the basis for longitudinal assessments of therapy outcomes for patients, such as those with epilepsy where objective identification of patient seizure activity and rhythms might help guide therapy optimization. The system is configurable for other disease states such as Parkinson’s disease and mood disorders.
